# Author Correction: Oil palm expansion and deforestation in Southwest Cameroon associated with proliferation of informal mills

**DOI:** 10.1038/s41467-021-22418-3

**Published:** 2021-04-09

**Authors:** Elsa M. Ordway, Rosamond L. Naylor, Raymond N. Nkongho, Eric F. Lambin

**Affiliations:** 1 Department of Earth System Science, Stanford University, 473 Via Ortega, Stanford, CA 94305 United States; 2 Department of Organismic and Evolutionary Biology, Harvard University, 26 Oxford Street, Cambridge, MA 02138 United States; 3 Stanford Woods Institute for the Environment, Stanford University, 473 Via Ortega, Stanford, CA 94305 United States; 4 Center on Food Security and the Environment, Stanford University, 616 Serra Street C100, Stanford, CA 94305 United States; 5 Department of Agronomic and Applied Molecular Sciences, University of Buea, Buea, Cameroon; 6 Georges Lemaître Earth and Climate Research Centre, Earth & Life Institute, Université catholique de Louvain, Place L. Pasteur 3, Louvain-la-Neuve, 1348 Belgium

**Keywords:** Environmental impact, Sustainability

Correction to: *Nature Communications* 10.1038/s41467-018-07915-2, published online 10 January 2019.

The original version of this Article contained an error in Fig. 5, in which the key labels for the lines representing ‘Inside concession’ and ‘Outside concession’ were mislabelled. The correct version of Fig. 5 is:

which replaces the previous incorrect version: 

This has been corrected in both the PDF and HTML versions of the Article.

