# Supplementary material for: Oil palm expansion and deforestation in Southwest Cameroon associated with proliferation of informal mills
Source: Nat Commun. 2019 Jan 10;10:114. doi: 10.1038/s41467-018-07915-2 (PMC6328567; doi:10.1038/s41467-018-07915-2)
Supplement: Supplementary file 1 — Supplementary Information [file 41467_2018_7915_MOESM1_ESM.pdf]

Supplementary Information

Oil palm expansion and deforestation in Southwest Cameroon associated with proliferation of informal mills

Ordway *et al.*

SUPPLEMENTARY METHODS

Oil palm classification accuracy. Random forest model results yielded an area-weighted accuracy of 94% (95% CI [0.92, 0.96]) for 2015 imagery classification and 95% (95% CI [0.93, 0.97]) for the year 2000 (**Fig. 3, Supplementary Table S2**). We were able to spectrally separate mature oil palm with an area-weighted user's accuracy of 91% in 2015 and 85% in 2000, and area-weighted producer's accuracies of 84% and 81%. Immature monoculture systems were classified with 87% and 74% user's accuracies in 2015 and 2000, and 80% and 83% producer's accuracies.

Spatial model prediction accuracy. To evaluate how well each spatial autocovariate model performed, based on predictive accuracy, we calculated the area under the receiver operating characteristic (ROC) curve (AUC) using hold-out sets from the 10-fold cross validation. The average AUC value for all cross-validation hold-out sets was sufficiently strong to lend confidence in both the oil palm expansion model (AUC = 0.8013, McFadden's R^2 = 0.25) and deforestation model (AUC = 0.7364, McFadden's R^2 = 0.13). ROC curves from all ten hold-out test sets for each model are illustrated in **Supplementary Figure S4**.

SUPPLEMENTARY FIGURES

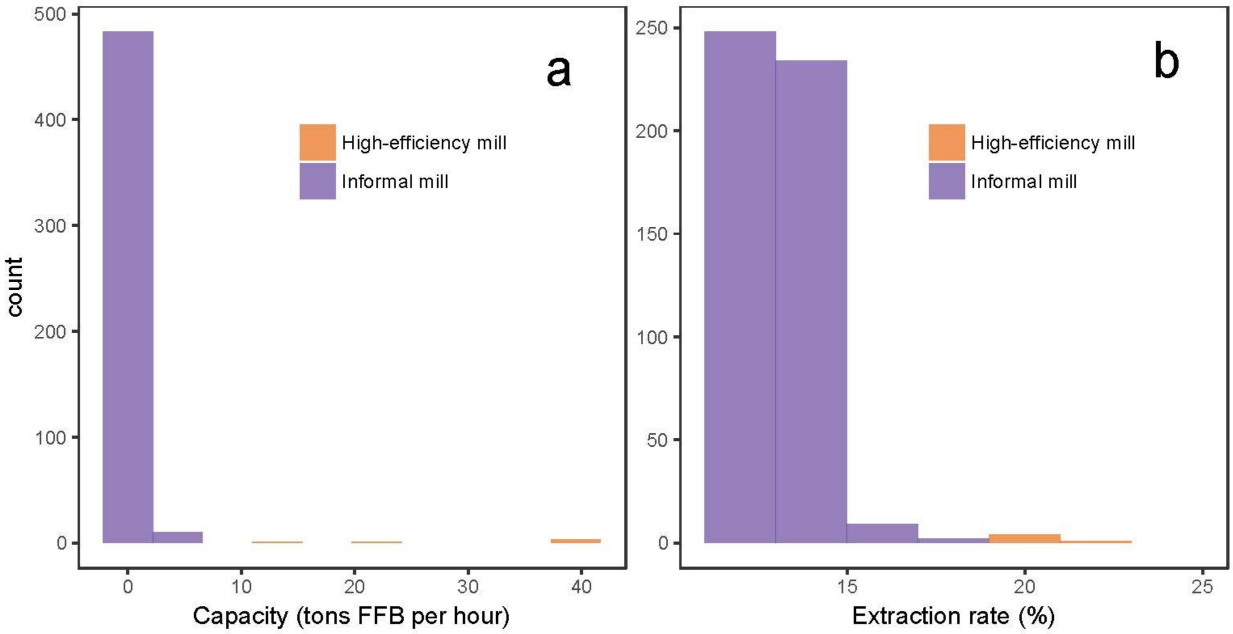

Supplementary Figure 1. Palm oil mill capacities and extraction rates. (**a-b)** Capacity (**a**) and extraction rate (**b**) frequency distributions for all mills mapped in the Southwest Region of Cameroon. High-efficiency agro-industrial mills (orange) exceeded all non-industrial mills (purple) in terms of both metrics.

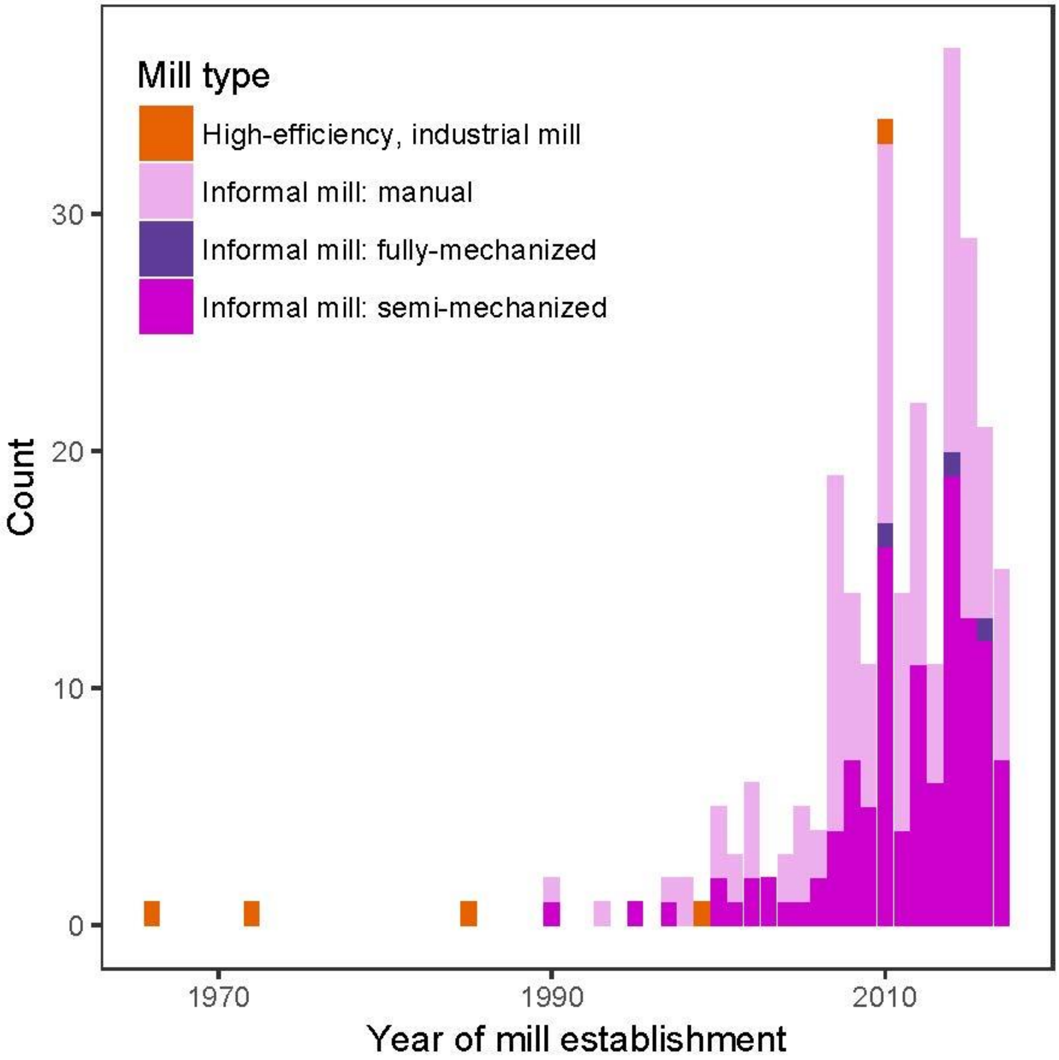

Supplementary Figure 2. Mill establishment. Year of establishment for all mills mapped in Southwest Cameroon. Only one of the five agro-industrial mills was built after the year 2000. Of the 498 mills mapped, 266 owners reported the year the mill was established. Over 95% (*n* = 255) of the mills with a recorded year of establishment were built in the year 2000 or later. Nearly all informal mills were manual or semi-mechanized systems.

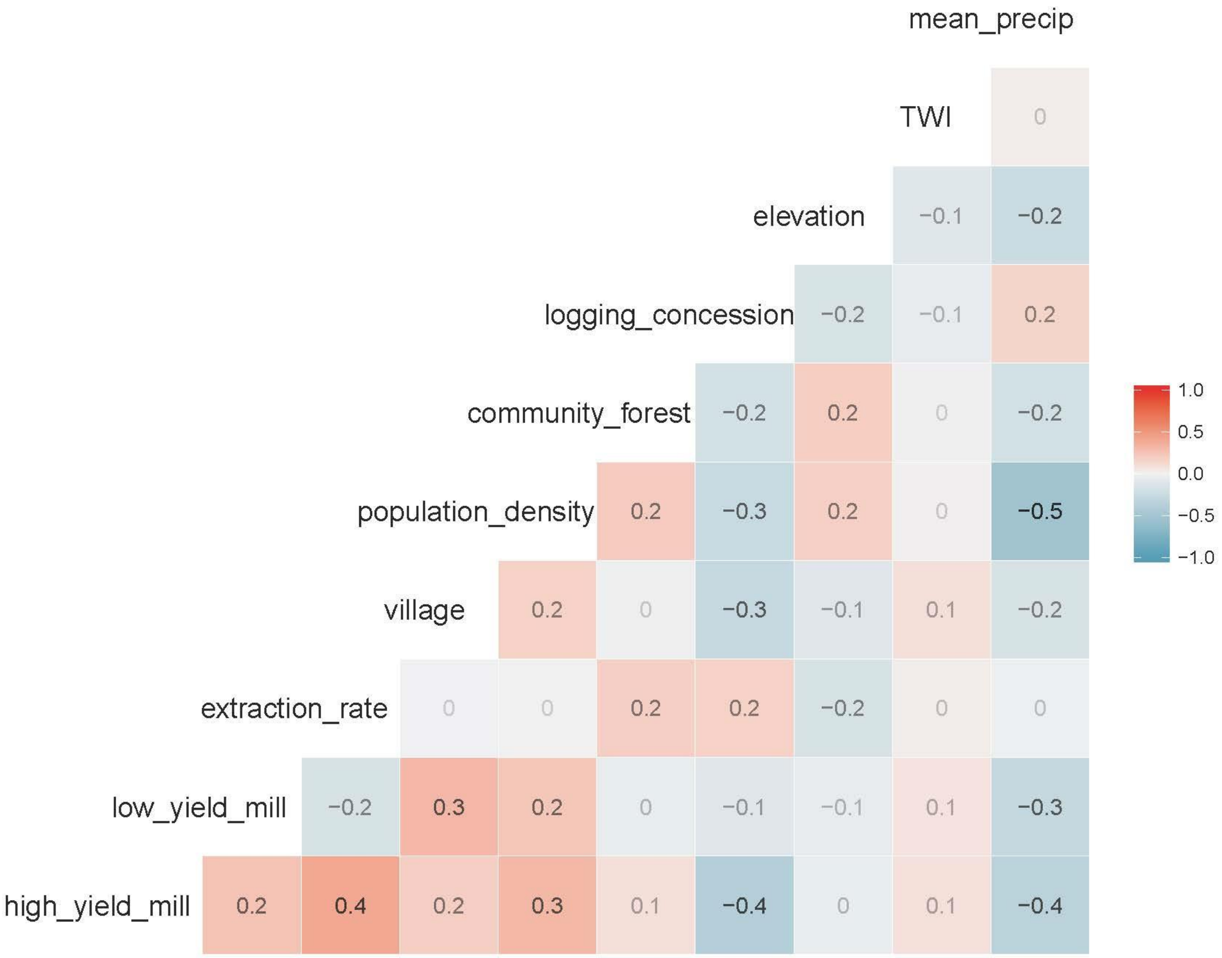

Supplementary Figure 3. Logit model variable covariance. Pearson's correlation coefficients for variables included in the expansion and deforestation binomial models as a test for multicollinearity. All correlation values were less than or equal to 0.5.

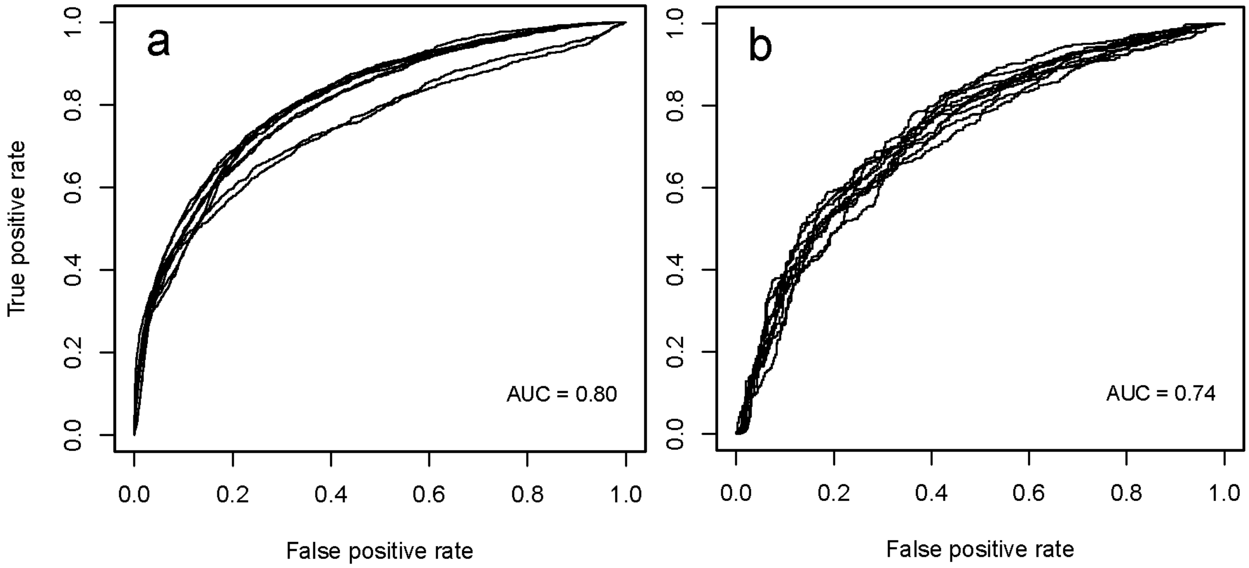

Supplementary Figure 4. Binomial logit model accuracy. (a-b) Receiving operator characteristic (ROC) curve for all ten cross validation test sets for the spatial autocovariate binomial logit models of oil palm expansion (**a**) and deforestation (**b**). Predictive accuracy, calculated as the average area under the ROC curve (AUC), was sufficiently strong to lend confidence in both models.

SUPPLEMENTARY TABLES

Supplementary Table 1. Oil palm expansion and deforestation spatial autocovariate binomial logit model results.

	Oil palm expansion				Deforestation due to oil palm expansion			
**Predictor variable**	**Estimate**	**Robust SE**	**OR**	**95% CI**	**Estimate**	**Robust SE**	**OR**	**95% CI**
Intercept	0.4344	0.3966	1.54	[0.71, 3.36]	-0.0484	0.4937	0.95	[0.36, 2.51]
Concession dummy variable	-1.3540**	0.2716	0.26	[0.15, 0.44]	-3.1533**	0.3734	0.04	[0.02, 0.09]
Distance to informal mill (km)	-0.0975**	0.0059	0.91	[0.90, 0.92]	0.1038**	0.0098	1.11	[1.09, 1.13]
Distance to high-efficiency mill (km)	-0.0053	0.0006	0.99	[0.99, 1.00]	-0.0179**	0.0013	0.98	[0.98, 0.98]
Mill extraction rate (%)	-0.0411**	0.0131	0.96	[0.94, 0.98]	-0.0470*	0.0200	0.95	[0.92, 0.98]
Distance to village (km)	-0.1104**	0.0094	0.90	[0.88, 0.91]	0.0796**	0.0132	1.08	[1.06, 1.11]
Population density (log people ha^{-1})	0.0955**	0.0208	1.10	[1.06, 1.15]	-0.3549**	0.0281	0.70	[0.66, 0.74]
Distance to community forest (km)	0.0065	0.0022	1.01	[1.00, 1.01]	-0.0259**	0.0029	0.97	[0.97, 0.98]
Elevation (m)	-0.0004	0.0001	1.00	[1.00, 1.00]	-0.0006	0.0001	1.00	[1.00, 1.00]
Topographic wetness index	0.0097	0.0115	1.01	[0.99, 1.03]	-0.1463**	0.0154	0.87	[0.84, 0.89]
0.1038 Mean annual precipitation (mm yr^{-1})	-0.0006	0.0001	1.00	[1.00, 1.00]	0.0009	0.0001	1.00	[1.00, 1.00]
Spatial autocovariate (x,y)	0.0981**	0.0005	1.10	[1.10, 1.10]	0.0005	0.0000	1.00	[1.00, 1.00]
Informal mill : Concession	0.0171	0.0121	1.02	[0.99, 1.04]	-0.1464**	0.0205	0.86	[0.83, 0.90]
High-efficiency mill : Concession	0.0088	0.0020	1.01	[1.00, 1.01]	0.0326**	0.0025	1.03	[1.03, 1.04]
Mill extraction rate : Concession	0.1642**	0.0181	1.18	[1.14, 1.22]	0.1898**	0.0253	1.21	[1.15, 1.27]

** β significant at Wald test, $p < 0.001$ and the odds ratio 95% CI does not overlap with 1.

* β significant at Wald test, $p < 0.05$ and the odds ratio 95% CI does not overlap with 1.

OR = odds ratio

Supplementary Table 2. Random forest land cover classification accuracy assessments.

2000 RF classification	Immature monoculture	Forest	Mature oil palm	Other	Total	Area-weighted User Accuracy (C_i)
Immature monoculture	70	0	2	23	95	74%
Forest	0	284	5	6	295	96%
Mature oil palm	0	9	81	6	96	85%
Other	12	9	8	359	388	93%
Total	82	302	96	394	874	
Area-weighted Producer Accuracy (O_j)	83%	99%	81%	88%		
Area-weighted Overall Accuracy = 95% (95% CI [0.93, 0.97])						

2015 RF classification	Immature monoculture	Forest	Mature oil palm	Other	Total	Area-weighted User Accuracy (C_i)
Immature monoculture	127	2	0	13	142	87%
Forest	1	251	7	4	263	95%
Mature oil palm	0	8	85	0	93	91%
Other	23	6	5	395	429	91%
Total	151	267	97	412		
Area-weighted Producer Accuracy (O_j)	80%	99%	84%	90%		
Area-weighted Accuracy = 94% (95% CI [0.92, 0.96])						

Supplementary Table 3. Binomial logit model variables

Response variable	Proportion*	Description
Oil palm expansion	1) 99.36%<br>2) 00.64%	Binomial: 1) no oil palm expansion occurred between 2000-2015, 2) oil palm expansion occurred between 2000-2015.
Deforestation due to oil palm expansion	1) 66.13%<br>2) 33.87%	Binomial: 1) forest converted to oil palm (2000-2015), 2) other land cover type converted to oil palm, e.g., other crop type (2000-2015).
**Predictor variable**	**Mean ± SD**	**Description**
Concession dummy variable	1) 95.37%<br>2) 4.63%	Binomial: 1) grid cells outside agro-industrial concession boundaries, 2) grid cells inside agro-industrial concession boundaries. Calculated using data from [61].
Distance to informal mill (km)	10.04 ± 7.77	Grid cell distance to low-efficiency, informal palm oil processing mill.
Distance to high-efficiency mill (km)	53.54 ± 35.26	Grid cell distance to high-efficiency, agro-industrial palm oil processing mill.
Mill extraction rate (%)	13.37 ± 1.61	Self-reported palm oil extraction rate, indicating the fraction of oil recovered from the quantity of fresh fruit bunches processed.
Distance to village (km)	5.11 ± 4.21	Grid cell distance to nearest village, town, or city. Calculated using data from [61].
Population density (log people ha^{-1})	0.32 ± 5.88	Log transformed, number of people ha^{-1}, sourced from [62].
Distance to community forest (km)	16.43 ± 9.54	Grid cell distance to community forest area. Calculated using data from [61].
Elevation (m)	378.80 ± 412.72	Gridded elevation, sourced from [66].
Topographic wetness index	10.23 ± 1.49	A steady-state wetness index calculated using [66]. Higher values represent drainage depressions, and lower values represent crests and ridges.
Mean annual precipitation (mm yr^{-1})	2938 ± 256.09	Mean annual precipitation, calculated for the years 1981-2016 using data from [49].

* Proportion of total grid cells included in model. Expansion model: n = 1,929,816; Deforestation model: n = 12,266.

Supplementary Table 4. Oil palm expansion and deforestation non-spatial binomial logit model results

	Oil palm expansion				Deforestation due to oil palm expansion			
**Predictor variable**	**Estimate**	**Robust SE**	**OR**	**95% CI**	**Estimate**	**Robust SE**	**OR**	**95% CI**
Intercept	2.5626**	0.2282	12.97	[8.29, 20.28]	-0.5013	0.4975	0.61	[0.23, 1.61]
Concession dummy variable	-1.9639**	0.1700	0.14	[0.10, 0.20]	-3.0402**	0.3759	0.05	[0.02, 0.10]
Distance to informal mill (km)	-0.1089**	0.0039	0.90	[0.89, 0.90]	0.0915**	0.0104	1.10	[107, 1.12]
Distance to high-efficiency mill (km)	-0.0063	0.0004	0.99	[0.99, 0.99]	-0.0240**	0.0011	0.98	[0.97, 0.98]
Mill extraction rate (%)	-0.0489**	0.0091	0.95	[0.94, 0.97]	-0.0489*	0.0200	0.95	[0.92, 0.98]
Distance to village (km)	-0.1081**	0.0061	0.90	[0.89, 0.91]	0.0773**	0.0133	1.08	[1.05, 1.11]
Population density (log people ha^{-1})	0.1201**	0.0130	1.13	[1.10, 1.16]	-0.3147**	0.0269	0.73	[0.69, 0.77]
Distance to community forest (km)	0.0113	0.0014	1.01	[1.01, 1.01]	-0.0264**	0.0029	0.97	[0.97, 0.98]
Elevation (m)	-0.0004	0.0000	1.00	[1.00, 1.00]	-0.0007	0.0001	1.00	[1.00, 1.00]
Topographic wetness index	0.0152	0.0074	1.02	[1.00, 1.03]	-0.1402**	0.0152	0.87	[0.84, 0.90]
Mean annual precipitation (mm yr^{-1})	-0.0003	0.0001	1.00	[1.00, 1.00]	0.0014	0.0001	1.00	[1.00, 1.00]
Informal mill : Concession	0.0092	0.0075	1.01	[0.99, 1.02]	-0.1526**	0.0211	0.86	[0.82, 0.89]
High-efficiency mill : Concession	0.0098	0.0012	1.01	[1.01, 1.01]	0.0291**	0.0026	1.03	[102, 1.03]
Mill extraction rate : Concession	0.2219**	0.0116	1.25	[1.22, 1.28]	0.1868**	0.0253	1.21	[1.15, 1.27]

** β significant at Wald test, $p < 0.001$ and the odds ratio 95% CI does not overlap with 1.
* β significant at Wald test, $p < 0.05$ and the odds ratio 95% CI does not overlap with 1.
OR = odds ratio
